# Influence of the rotation angles on the mechanical behavior of the one reci instrument

**DOI:** 10.1038/s41405-025-00327-7

**Published:** 2025-04-12

**Authors:** Renaud Giess, Éric Mortier, Marie Delanoë, Romain Hocquel, Jean-Marc Martrette, Rémy Balthazard, Marin Vincent

**Affiliations:** 1 Départment de dentisterie restauratrice et endodontie, Faculté d’odontologie de Lorraine, Université de Lorraine, F-54000 Nancy, France; 2 ERPI, Université de Lorraine, F-54000 Nancy, France; 3 CNRS, IJL, Université de Lorraine, F-54000 Nancy, France; 4 Faculté d’odontologie de Lorraine, Université de Lorraine, F-54000 Nancy, France; 5 Faculté de médecine, EA 3450, Université de Lorraine, F-54000 Nancy, France; 6 CNRS, LEM3, Université de Lorraine, F-57000 Metz, France

**Keywords:** Endodontic files, Root canal treatment

## Abstract

**Aims:**

The reciprocity is a complex endodontic kinematic involving many parameters. The most important of these is undoubtedly the selection of clockwise and counterclockwise rotation angles. In this context, the aim of this study was to determine the influence of clockwise and counterclockwise rotation angles on the mechanical properties of 25/0.06 One Reci reciprocity instruments.

**Method:**

For this purpose, 5 groups of 10 25/0.06 One Reci instruments were used and each group was associated with a pair of counterclockwise/clockwise rotation angles (CCW/CW). In order to study only one variable at a time, one of the two angles was fixed and the second was increased or decreased. The distribution of angles was as follows: Group 1: 170°/60°; Group 2: 150°/60°; Group 3: 170°/30°; Group 4: 170°/90°; Group 5: 210°/60°. Thanks to a load/unload endodontic protocol carried out on a tensile bench, we quantified for each tested pair of angles (i) the cutting efficiency, (ii) the screwing effect and (iii) the generated torque.

**Results:**

Increasing or decreasing one of the two rotation angles influences the mechanical behavior of the instruments, as does the resulting range. Therefore, our results showed a direct influence of rotation angles on the mechanical behavior of endodontic instruments.

**Conclusion:**

This study analyzes the influence of clockwise and counterclockwise rotation angles on the mechanical properties of 25/0.06 One Reci reciprocity instruments. The results of this work tend to demonstrate a direct influence of rotation angles on the mechanical behavior of 25/0.06 One Reci instruments.

## Introduction

The latest endodontic major advancement is based on the mechanization of NiTi instruments in the mid-90s, the continuous rotation instrumental kinematic making the procedure safer and more reproducible [1–10]. More than 10 years after the arrival of continuous rotation, a new instrumental dynamic is shaking up the protocols of canal shaping. This new instrumental kinematic, called reciprocity, is an asymmetrical reciprocating movement involving an alternation of counterclockwise (CCW) and clockwise (CW) rotation angles. However, the reciprocating motion is more complex, involving a large number of parameters. In addition to the speed and rotation angles, other settings generally not communicated by the manufacturers, such as CCW and CW acceleration and deceleration, torque or standstill time at each change of direction, make these movements complex to analyze.

According to the scientific literature, this reciprocating motion offers several advantages such as the respect of canal trajectory [11], the elimination of debris as well as the reduction of dentinal microcracks [12]. This instrumental kinematic is especially advantageous to reduce cyclic fatigue and torsional stress [13, 14].

A large number of researches concerning the mechanical properties of instruments in terms of flexibility, cyclic fatigue, resistance to torsion, and separation incidence have been carried out [15–32]. Heat treatment is one of the fundamental approaches used to modify the crystallographic arrangement of NiTi endodontic files [33–41], and thus their mechanical behaviors [42–47]. Many studies have compared endodontic files from different brands with various designs, kinematics, and heat treatment technologies [45–48]. All these studies showed mechanical improvement of heat treated endodontic files but highlight that many factors can influence mechanical resistance, each parameter influencing all the other ones. Therefore, for a fundamental approach, it seems important to test only one parameter at a time.

Among all these mechanical property researches, very few articles have directly focused on the influence of clockwise and counterclockwise rotation angles on the mechanical properties of reciprocity instruments. Reciprocity instruments generally have a left-handed pitch and their active angle, the largest, always goes in the pitch direction. Therefore, CCW angles give the instrument cutting efficiency, but also its screwing effects, while CW angles allow discharging the torsional stresses accumulated during cutting. The range between the two angles is also important and determines the ratio between cutting efficiency, screwing effect and discharge of torsional stresses. However, two reciprocity instruments can have a similar range, but different CCW and CW angles, leading to very different [49–53].

The present study aims to analyze the effect of different asymmetrical reciprocating rotation angles on the cutting efficiency and screwing effects of the One Reci instrument (MicroMega, Besançon, France). The hypothesis of this study is that a single reciprocating setting could not be suitable for all clinical situations. A better understanding of this kinematic would certainly improve the conduct and reproducibility of endodontic treatments. In this perspective, several CCW and CW rotation angle pairs were evaluated during a load/unload endodontic protocol allowing for the recording of cutting efficiency and screwing effects, respectively. Simultaneously, the torque required for reciprocating motion was measured.

## Materials and methods

### Resin blocs

**Fig. 1:**
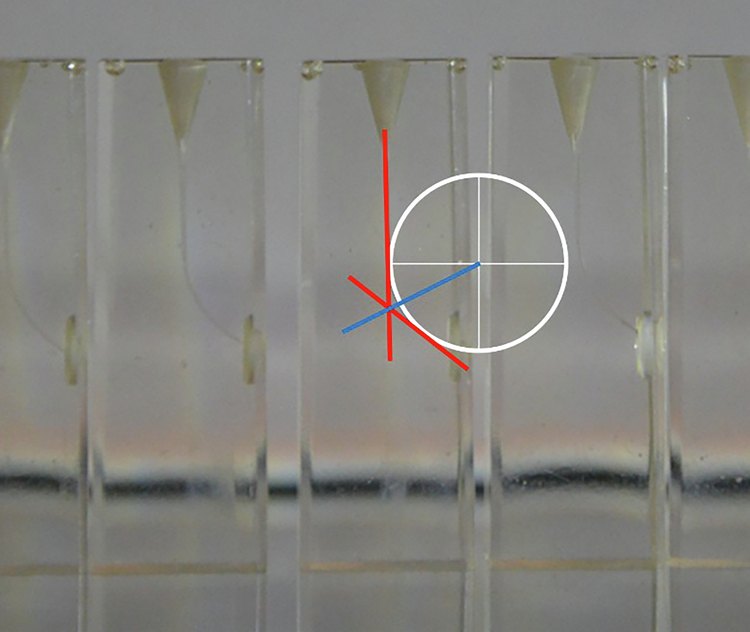
The used endodontic resin blocks. The curvature angle and the radius of curvature are determined by the red and blue lines, respectively.

5 groups of 10 J shape resin blocks (Dentsply Sirona, Ballaigues, Switzerland) were employed for the performance and security tests. The used resin blocks present an average length of 18 mm, an apical permeability, and an average radius of curvature of 4.5 mm (Fig. 1). These endodontic blocks mimic challenging endodontic situations, according to the Endodontic Case Difficulty Assessment Form and Guidelines edited by the American Association of Endodontic.

### Endodontic files

5 groups of 10 25/0.06 One Reci instruments were used in this study. Table 1 shows the used endodontic protocol. Each group was associated with a pair of counterclockwise/clockwise rotation angles (CCW/CW). In order to study only one variable at a time, one of the two angles was fixe and the second was increased or decreased. All angle pairs are identified in CCW/CW direction. The distribution of angles was as follows: Group 1: 170°/60°; Group 2: 150°/60°; Group 3: 170°/30°; Group 4: 170°/90°; Group 5: 210°/60°.

**Table 1:** Shaping protocol used for the endodontic tests.

Step	Instrument*	Working length	Kinematic parameters	Action
1	K. 10 file	18 mm		Root canal exploration + permeabilization
2	K.10 file	–	–	Permeabilization
3	One G instrument	18 mm	350 rpm 1.2 N.cm	Glide path
4	K.10 file	–	–	Permeabilization
5	Single use One RECI instrument	18 mm	170°/60° 150°/60° 170°/30° 170°/90° 210°/60°	Shaping
6	K.10 file	–	–	Permeabilization

All instruments were tested following a load/unload endodontic protocol.

Before each test, the resin blocks were explored using a K.10 manual file for reproducibility purposes. In order to respect the clinical protocol of the One Reci instruments, a glidepath with a 14/0.03 One G (MicroMega, Besançon, France) instrument was used before testing the One Reci instruments.

### Load/unload endodontic protocol

**Fig. 2:**
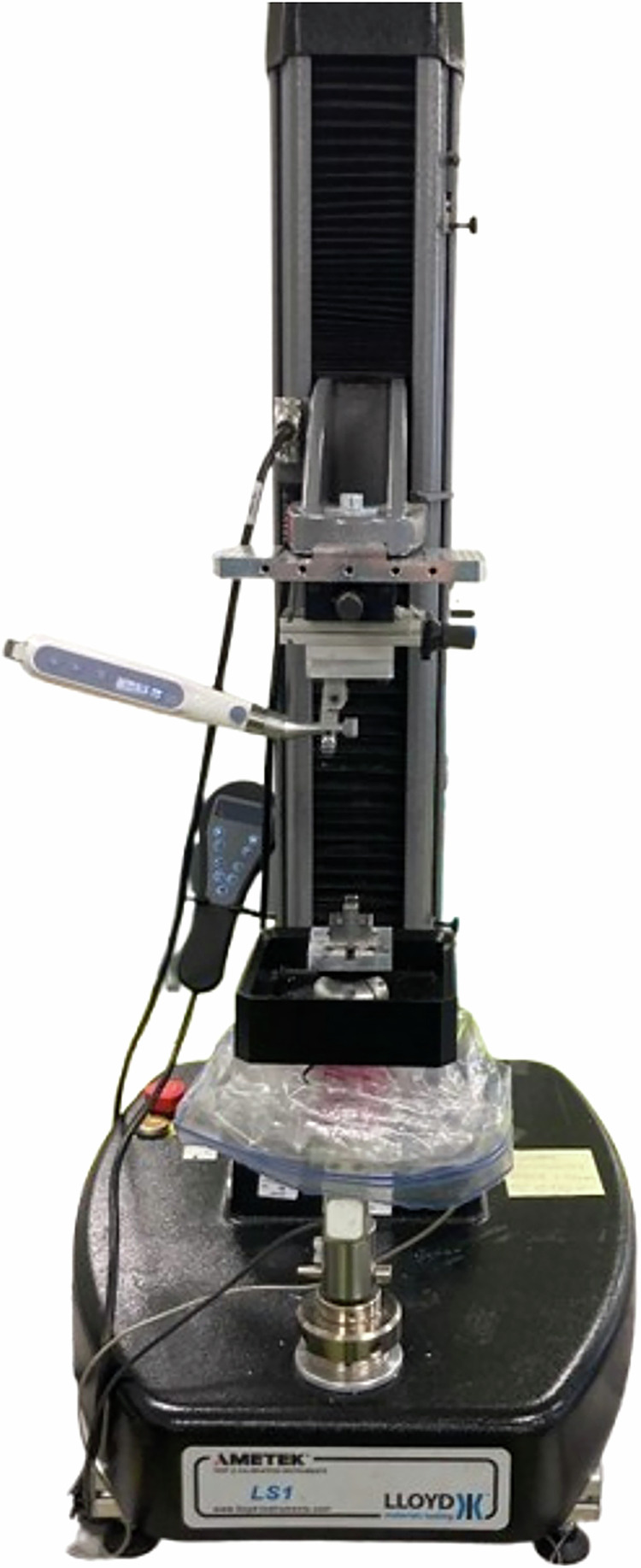
Tensile bench and endodontic assembly used for this study.

The load/unload endodontic protocol, allowing respectively downward/upward pecking motions, was carried out on a LS1 tensile bench (Ametek Lloyd Instruments Ltd., Bognor Regis, UK) controlled by the NexygenPlus software version 4.1.1.829 (Ametek, Berwyn, USA) in the dental engineering technical hall of the faculty of odontology of Lorraine (Campus Brabois Santé, University of Lorraine, France) (Fig. 2).

The tensile bench is composed of a platform on which the resin block is located. This platform is associated with a S2M/50 N load cell with an output signal of 2 mV/V (HBM, Darmstadt, Germany) and a CS1211-B 0.3Nm torque sensor (TE Connectivity Measurement Specialties, Galway, Irland). The endodontic instrument is insert on an endodontic contra-angle connected to a programmable endodontic motor (Dual Move®, MicroMega, Besançon, France). The endodontic motor is connected to the column and carries out the up-and-down pecking motions required by the protocol. Thanks to the micrometric screws, the axis of the tested endodontic instrument can be perfectly aligned with that of the resin block canal. The instrument under test is positioned from the occlusal edge of the resin block using an automatic detection tool.

The protocol corresponds to a free load/unload test involving 25 successive cycles, divided into 9 groups of cycles, allowing the up-and-down pecking motion of the tested endodontic instrument in a resin block [54]. The instrumental pressure was kept constant and reproducible thanks to our experimental protocol obtained by the association of (i) a constant up-and-down pecking speed controlled by the bench, (ii) a constant instrumental kinematics given by the same endodontic motor and (iii) a machining of the same resin blocks with (iv) the same instruments. Therefore, this protocol made it possible to directly analyze the instrumental cutting efficiency and screwing effects. Changing a parameter, here the rotation angles and thus the instrumental kinematics, allows testing its influence on the instrumental cutting efficiency and screwing effects during the shaping of the resin block.

Complete instrumental withdrawal is performed between each cycle allowing (i) a canal irrigation with 1 mL of 96% ethanol to remove debris and (ii) a verification of the apical patency. This protocol was established to be reproducible. To mimic the clinical reality, three groups of parameters appropriate to the work of the associated canal section were determined as follow:

- Canal penetration from 10 to 14 mm, before the curvature (Table 2a),
- Canal penetration from 14 to 16 mm, in the coronal part of the curvature (Table 2b),
- Canal penetration from 16 to 18 mm, in the apical part of the curvature (Table 2c).

**Table 2:** a Load/unload endodontic protocol: penetration level from 10 to 14 mm; b Load/unload endodontic protocol: penetration level from 14 to 16 mm; c Load/unload endodontic protocol: penetration level from 16 to 18 mm.

a					
			Penetration [mm]	Speed [mm/s]	Stop [s]
Group 1	Cycle 1	Load	10 9.5	5 5	
		Unload			
	Cycle 2	Load	10.5 10	5 5	
		Unload			
	Cycle 3	Load	11 10.5	5 5	
		Unload			
	Irrigation		−51	15	5
Group 2	Cycle 4	Load	11.5 11	5 5	
		Unload			
	Cycle 5	Load	12 11.5	5 5	
		Unload			
	Cycle 6	Load	12.5 12	5 5	
		Unload			
	Irrigation		−51	15	5
Group 3	Cycle 7	Load	13 12.5	5 5	
		Unload			
	Cycle 8	Load	13.5 13	5 5	
		Unload			
	Cycle 9	Load	14 13.5	5 5	
		Unload			
	Irrigation		−51	15	5

b					
			Penetration [mm]	Speed [mm/s]	Stop [s]
Group 4	Cycle 10	Load	14.25 14	2 2	
		Unload			
	Cycle 11	Load	14.5 14.25	2 2	
		Unload			
	Cycle 12	Load	14.75 14.5	2 2	
		Unload			
	Cycle 13	Load	15 14.75	2 2	
		Unload			
	Irrigation		−51	15	5
Group 5	Cycle 14	Load	15.25 15	2 2	
		Unload			
	Cycle 15	Load	15.5 15.25	2 2	
		Unload			
	Cycle 16	Load	15.75 15.5	2 2	
		Unload			
	Cycle 17	Load	16 15.75	2 2	
		Unload			
	Irrigation		−51	15	5

c					
			Penetration [mm]	Speed [mm/s]	Stop [s]
Group 6	Cycle 18	Load	16.25 16	2 2	
		Unload			
	Cycle 19	Load	16.5 16.25	2 2	
		Unload			
	Irrigation		−51	15	5
Group 7	Cycle 20	Load	16.75 16.5	2 2	
		Unload			
	Cycle 21	Load	17 16.75	2 2	
		Unload			
	Irrigation		−51	15	5
Group 8	Cycle 22	Load	17.25 17	2 2	
		Unload			
	Cycle 23	Load	17,5 17.25	2 2	
		Unload			
	Irrigation		−51	15	5
Group 9	Cycle 24	Load	17.75 17.5	2 2	
		Unload			
	Cycle 25	Load	18 17.75	2 2	
		Unload			
	Irrigation		−51	15	5

^*^irrigation with 96% ethanol irrigation is performed before and after each instrument pass to remove cutting debris.

The torque, the vertical components of the force and displacement are recorded at a rate of 26 Hz. At the end of each test, the maximum load and unload values are recorded for each group of cycles.

### Statistics

Numerical data were analyzed using non-parametric Kruskal-Wallis statistical tests with Dunn’s correction (α = 0.05) for multiple comparisons. The results were considered statistically significant for a *P* value < 0.05. All statistical analyzes were performed using GraphPad Prism® software (San Diego, California, US).

## Results

**Fig. 3:**
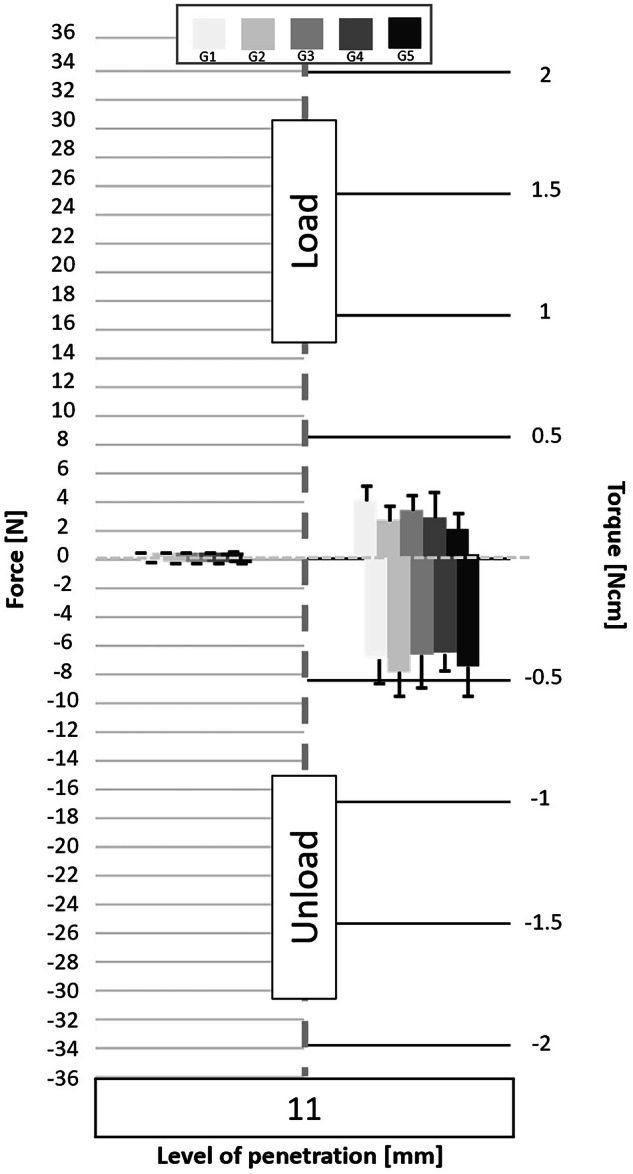
Graphs of the forces and torque obtained during the load/unload endodontic tests at a penetration level of 11 mm.

**Fig. 4:**
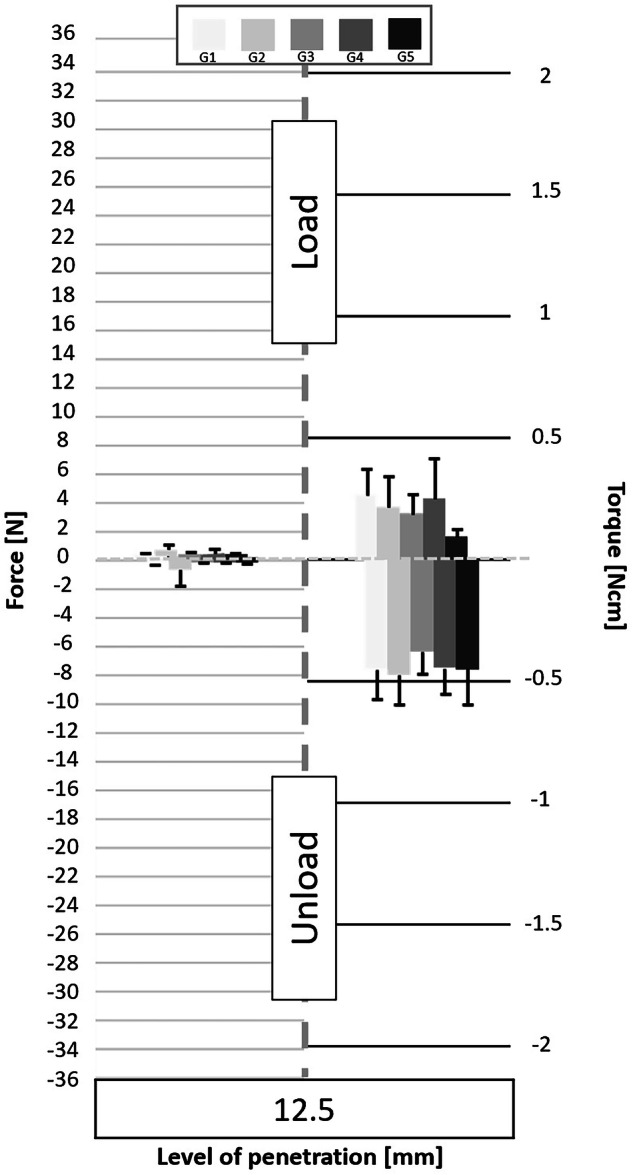
Graphs of the forces and torque obtained during the load/unload endodontic tests at a penetration level of 12.5 mm.

**Fig. 5:**
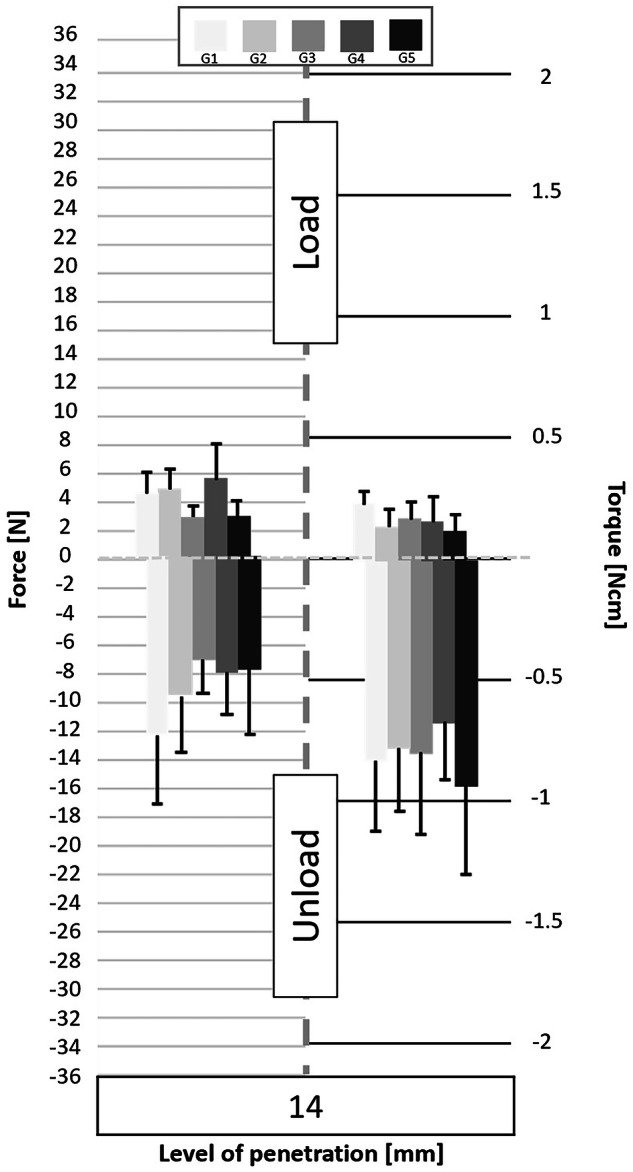
Graphs of the forces and torque obtained during the load/unload endodontic tests at a penetration level of 14 mm.

**Fig. 6:**
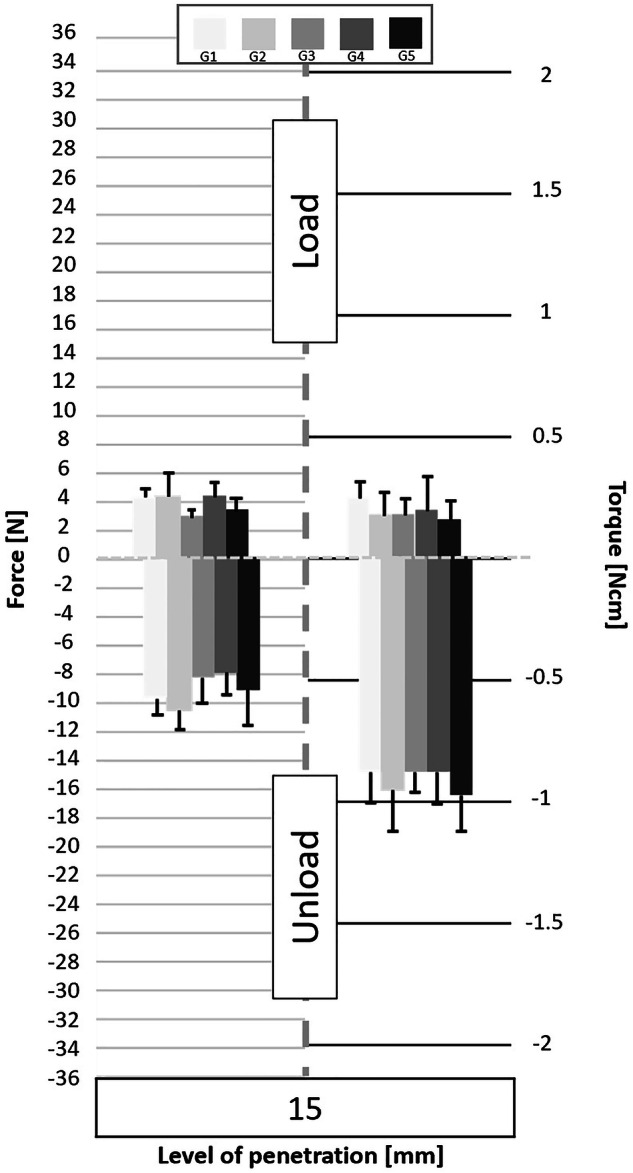
Graphs of the forces and torque obtained during the load/unload endodontic tests at a penetration level of 15 mm.

**Fig. 7:**
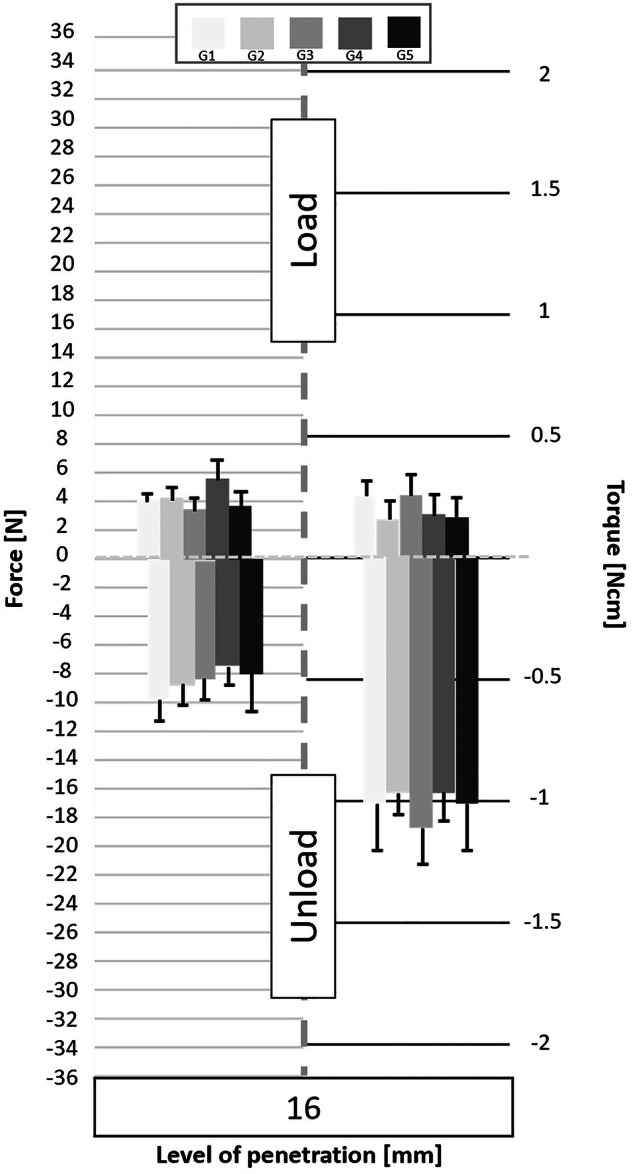
Graphs of the forces and torque obtained during the load/unload endodontic tests at a penetration level of 16 mm.

**Fig. 8:**
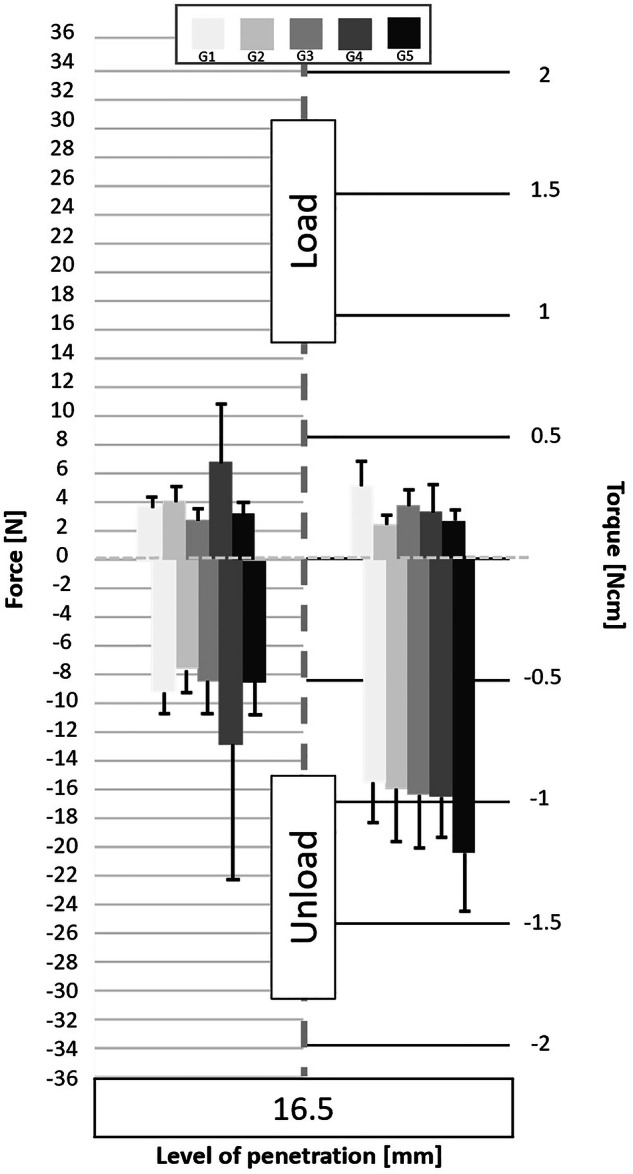
Graphs of the forces and torque obtained during the load/unload endodontic tests at a penetration level of 16.5 mm.

**Fig. 9:**
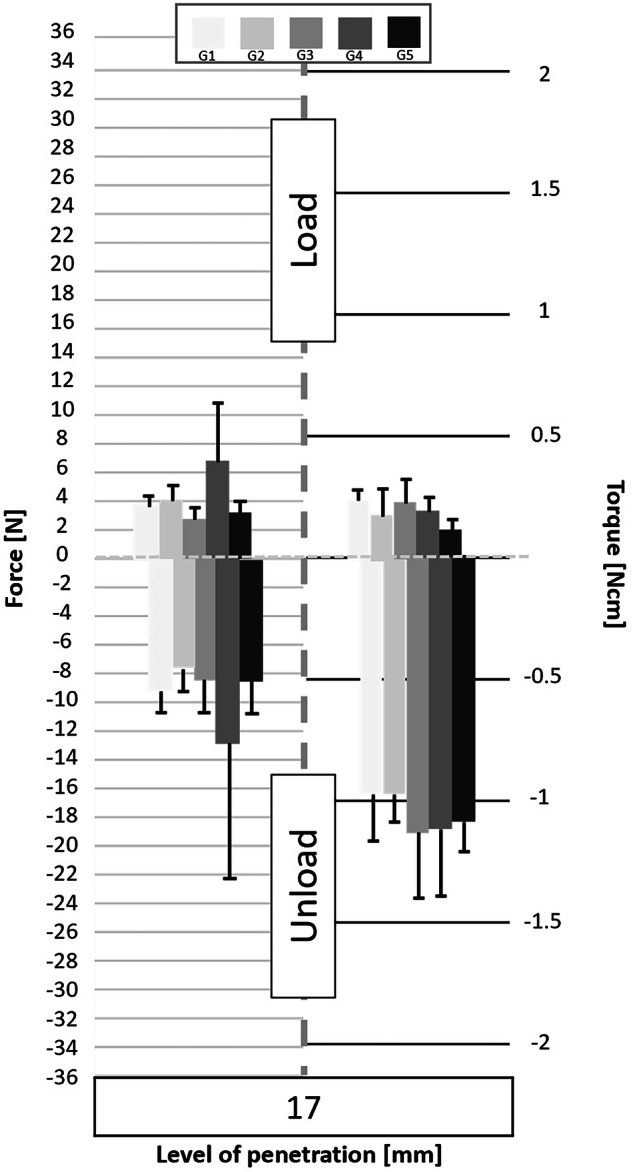
Graphs of the forces and torque obtained during the load/unload endodontic tests at a penetration level of 17 mm.

**Fig. 10:**
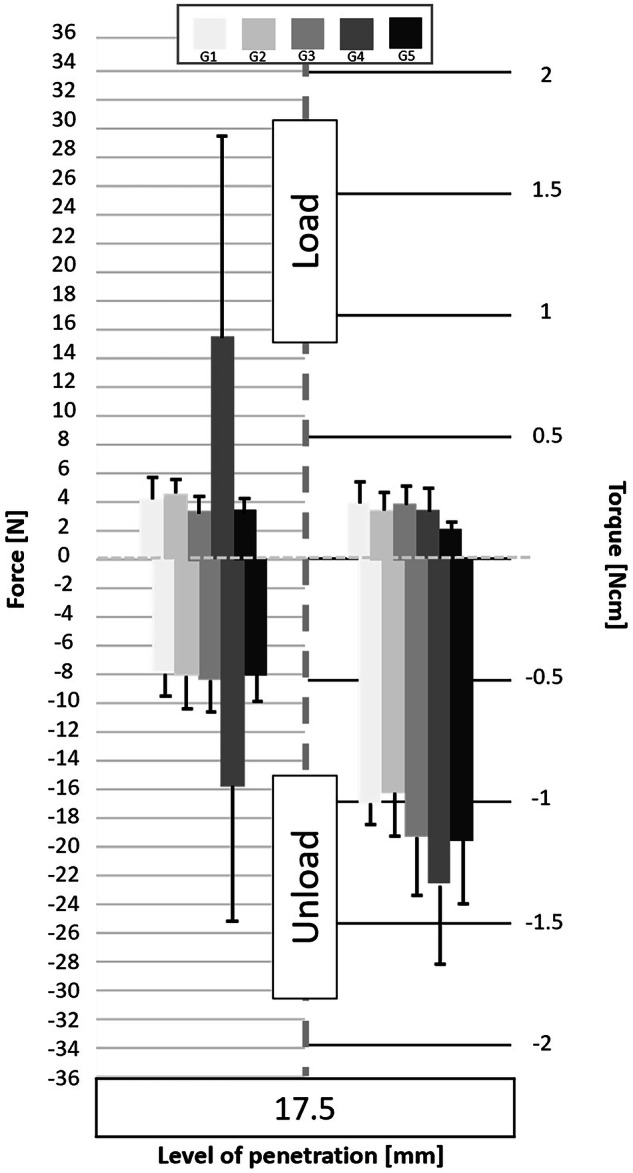
Graphs of the forces and torque obtained during the load/unload endodontic tests at a penetration level of 17.5 mm.

**Fig. 11:**
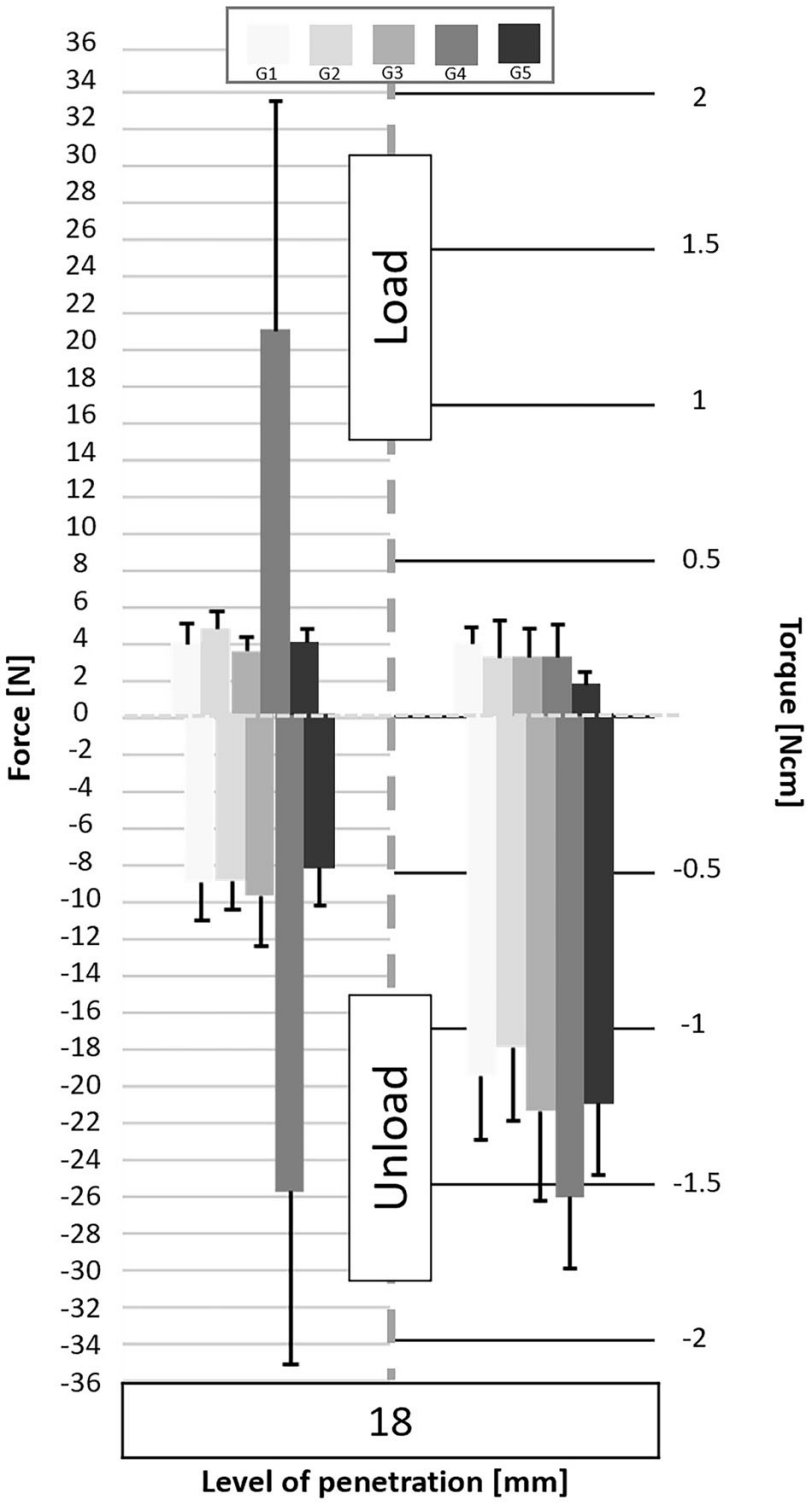
Graphs of the forces and torque obtained during the load/unload endodontic tests at a penetration level of 18 mm.

Following the processing of the data collected by the cell force, the minimum and maximum values of cycles were analyzed. Figures 3 to 11 show the load/unload forces and the associated load/unload torque of the different tested pairs of angles in function of the penetration level. The data on the graph are presented with means ± standard deviations. Results are presented in Tables 3a to 4b and detail respectively (i) the differences in the load/unload forces and (ii) the differences in the associated load/unload torque of the different pairs of rotation angles.

**Table 3:**
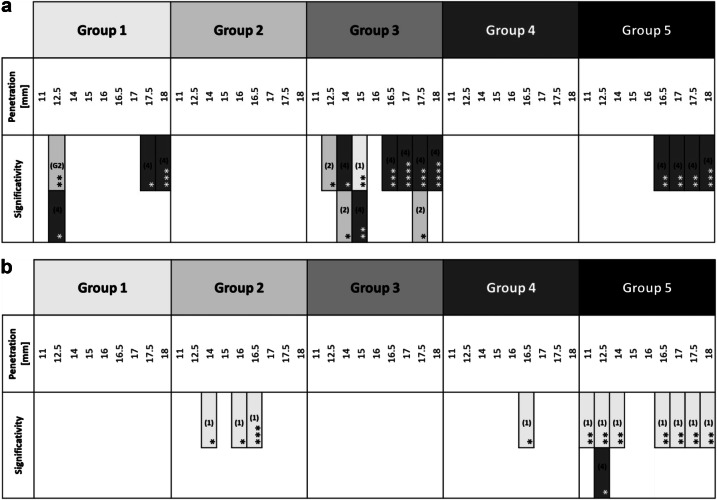
a Positive forces obtained during the downward pecking motion. The significant differences are in favor of the groups noted in the column; b Torques obtained during the downward pecking motions. The significant differences are in favor of the groups noted in the column.

Group 1: 170°/60°; Group 2: 150°/60°; Group 3: 170°/30°; Group 4: 170°/90°; Group 5: 210°/60°. *P < 0.05; **P < 0.01; ***P < 0.001; ****P < 0.0001.

**Table 4:**
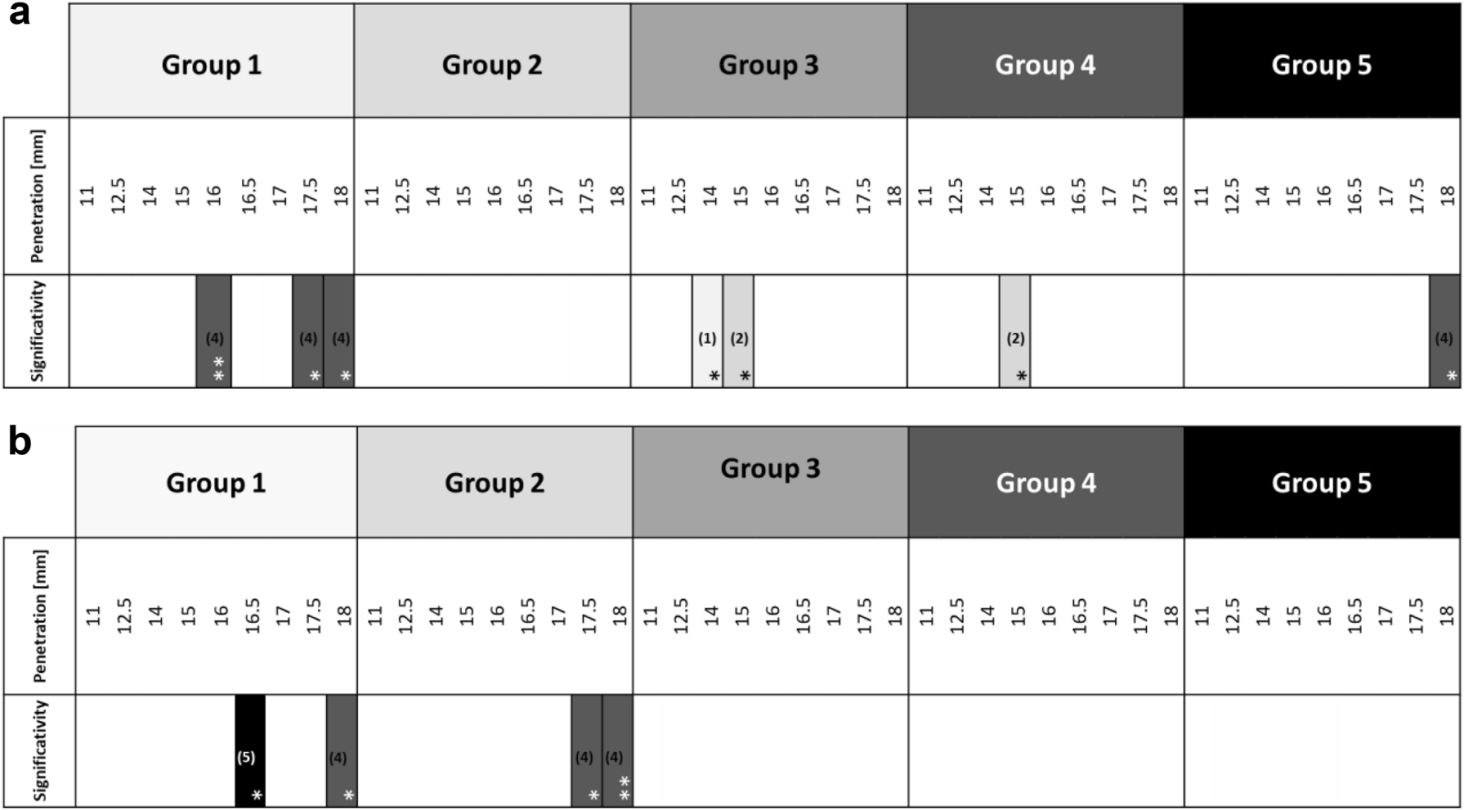
a Negative forces obtained during the upward pecking motions. The significant differences are in favor of the groups noted in the column; b Torques obtained during the upward pecking motions. The significant differences are in favor of the groups noted in the column.

Group 1: 170°/60°; Group 2: 150°/60°; Group 3: 170°/30°; Group 4: 170°/90°; Group 5: 210°/60°. *P < 0.05; **P < 0.01; ***P < 0.001; ****P < 0.0001.

### Loading time

The positive forces obtained during the downward pecking motions, corresponding to the loading times, essentially underline the cutting effect of the instruments. Several significant differences are found (Table 3a). The analysis of the load force results shows that:

- the G3 group obtains the best results;
- the G4 group obtains the worst results;
- groups G1, G2 and G5 do not highlight any significant differences; however, the results give the following trend: G5 > G1 > G2.

Torque represents the energy required by the endodontic motor to maintain a constant rotation speed regardless of the encountered constraint. During the loading times, the analysis of the torque results does not show any real difference (Table 3b). Nevertheless, group G5 obtains the lowest load torque values.

Therefore, it seems that:

- for a same CCW angle, a reduction in the CW angle improves the cutting efficiency (G3 is the best group);
- for a same CCW angle, an increase in the CW angle reduces the cutting efficiency (G4 is the worst group);
- the greater the difference between the two angles, the greater the cutting efficiency (G5 > G1 > G2);
- the greater the difference between the two angles, the lower the load torque (G5 > G2).

### Unloading times

The negative forces obtained during the upward pecking motions, corresponding to the unloading times, essentially highlight the screwing effects which oppose the withdrawal of the instrument. Some significant differences are found (Table 4a). The analysis of the unload force results shows that:

- the G4 group obtains the worst results in the apical region;
- the groups G1, G2, G3 and G5 do not highlight any significant differences except at 14 mm and 15 mm where G1 and G2 are significantly worst; however, the results give the following trend: G5 > G1, G2, G3, G4.
  During the unload times, the analysis of the torque results does not show any difference (Table 4b). The trends are:
- groups G1 and G2 obtain the lowest unload torque values;
- group G4 obtains the highest unload torque values in the last millimeter.

Therefore, it seems that:

- for the same CCW angle, an increase in the CW angle increases the screwing effect during maximum sheathing (G4 is the worst group);
- for the same CW angle, a reducing in the CCW cutting angle increases the screwing effect at the beginning of the curvature (G1 et G2 are the worst groups);
- for the same CW angle, an increase of range between the two angles linked to a strong increase of the CCW angle seems to slightly reduce the screwing effect (G5);
- for the same CW angle, an increase of the CCW angle increases the unload torque (G5 is the worst group).

## Discussion

While the literature describes resistance to cyclic fatigue and resistance to torsion as advantages of the reciprocating motion, apical debris extrusion or canal transportation, for example, seem to be more questionable [12]. These differences in conclusions are related to the complexity of analyzing the kinematics of the asymmetrical reciprocating motion [55, 56]. Each reciprocity instrument is only qualified for a given pair of angles and a given rotational speed, leaving the practitioner free to use it on the endodontic motor of it choice. Nevertheless, other settings, especially CCW and CW acceleration and deceleration, torque or standstill time at each change of direction, are generally not communicated nor configurable. These settings are directly linked to the ability of endodontic motors to be reproducible between them, while the literature highlights the opposite [57–59]. Therefore, in addition to its complex settings, this kinematic is dependent on the power of the rotating motor and their clinical usage [57, 60, 61]. All these reasons could explain the disparities found on the asymmetrical reciprocating motion kinematics in the literature, underlining the need for an evidence-based approach.

The aim of our study was to analyze the influence of the CCW/CW rotation angles on the mechanical behavior of the One Reci instrument. For this purpose, we quantified for each tested pair of angles (i) the cutting efficiency linked to the load forces, (ii) the screwing effect linked to the unload forces and (iii) the torque linked to the ability of the endodontic motor to maintain a constant rotation speed.

Based on the obtained load forces, we can classify the tested pair of angles as follows:

170°/30° (G3) > 210°/60° (G5) > 170°/60° (G1) > 150°/60° (G2) > 170°/90° (G4)

Load forces, linked to the cutting efficiency, increase as the instrument advances into the canal. However, load associated torques remain relatively stable throughout the preparation. Angle variations do not affect the load torque required to the progression of the instrument into the canal. Increasing the clockwise angles decreases the cutting capacity of the instrument, especially in the last apical millimeter when the instrument encounters high resistance (G4), while decreasing them increases the cutting capacity (G3) due to a higher number of rotation cycles per minute. The most important parameter is the difference between the two angles: the greater the range, the more effective the cut (G5). Nevertheless, according to Saber and al. (2013), the reduction of the reciprocity range will increase resistance to cyclic fatigue, allow better centering, decrease canal transport, but increase preparation [49]. Others studies confirmed that reducing the range of reciprocity, by decreasing the CCW angle, improves resistance to cyclic fatigue [50, 51]. Therefore, a high range linked to a high CCW rotation angle would improve the performance of the instrument, but would reduce the safety of use.

Based on the obtained unload forces, we can classify the tested pair of angles as follows:

210°/60° (G5) > 170°/60° (G1) - 150°/60° (G2) - 170°/30° (G3) > 170°/90° (G4)

Increasing the clockwise angles increases the unload forces link to screwing effects (G4), while an increase in the CCW angle decreases them (G5). Experimental conditions with a constant descent speed of the instrument in the canal, combined with the high CW angle (G4), do not allow for sufficiently rapid shaping of the canal, which promote tip binding. As a result, the unload forces required for it disengagement are increased Omori et al. [53]. compared the effect caused by an increase in the range of reciprocity between the angles 150°/30° and 240°/120° [53]. They found that the instrument with 240°/120° angles showed fewer screwing forces compared to it homologue with 150°/30° angles. Therefore, it would have been interesting to test an increase in overall range by increasing both the CW and CCW angles to validate these results with the One Reci instrument. Our results show that an increase of range linked to an CCW angle increase (G5) tend to decrease the screwing effects. Also, It would be interesting to validate this hypothesis during an increase in range linked to a decrease in CW angle.

Regarding torque, the load values are generally consistent regardless of the CCW/CW angle pairs and penetration levels. This could be explained by our experimental conditions, which involved only a same type of instrument, a same canal geometry, and a same load speed setting. However, the high 210° CCW angle of G5, providing better cutting efficiency and easier progression within the canal, could potentially explain its lower load torque values.

For the same reasons related to the experimental conditions, the consistency of torque values during unload can also be observed. However, the values obtained for group G4 in the last apical millimeter could be explained by its high 90° CW angle, causing tip binding and thus requiring higher unload torque to maintain rotation speed during disengagement. G1 and G2 achieve the best lowest unload torque values: the lower the range linked to a low CCW rotation angle, the lower the unload torque values. It would be interesting to observe if the same results would be obtained for a decrease of range linked to the CW angle.

All of these potential mechanical behavior improvements are achieved by forcing the endodontic motor into a configuration for which the One Reci instrument has not been qualified. To exploit all these data at once, it would be necessary to implement these scenarios in endodontic motors monitoring in real time the pressure forces and the torque undergone by the instrument to identify the best pair of angle configuration according to the clinical situations. Effectively, literature has shown that motors capable of adapting the kinematic of endodontic instruments according to the perceived constraints by the instruments make it possible to improve their mechanical behaviors. Thanks to this monitoring, the endodontic motor will adapt its movement to each clinical situation and improve the safety of endodontic treatment [61, 62].

With a fundamental approach, the main objective of our collection method was to change only one variable at a time and to fix all the other parameters. The use of a tensile bench made it possible to maintain a constant instrumental pressure through (i) a constant up-and-down pecking speed, (ii) the use of a single endodontic motor, (iii) a single type of endodontic simulator and (iv) a single type of instrument. This constant pressure allows the reproducible analyze of the instrumental cutting efficiency and screwing effects. Therefore, any modification of the instrumental settings allow to directly analyze its impact on these two mechanical behaviors. The fully automated and computerized system ensured the reproducibility of the data collection. Likewise, because the measured endodontic motors values differ from the manufacturers’ declared values [55, 56], the association of a torque sensor to the tensile bench allows reproducibility of torque data regardless of the tested motor. Another study highlights that these experimental conditions can be adapted to different motors and different kinematics [61]. However, we emphasize human intervention during the time of irrigation and verification of the apical patency. Therefore, a fully automated solution could make it possible to propose a reproducible method allowing an evidence-based approach in terms of cutting efficiency and screwing effects.

Finally, further studies are needed to extend these results to other endodontic reciprocating instruments. In addition, tests on extracted natural teeth could validate the obtained preclinical results. Indeed, the use of resin blocks allows better experimental standardization, but their mechanical properties differ from those of dental tissues [63].

## Conclusion

Within the limits of our study, the best way to increase the performance of the One Reci instrument seems to be:

- to reduce the CW rotation angle, thus promote the active movement, when high cutting efficiency is desired (G3);
- to increase the range linked to a high CCW rotation angle when a compromise between cutting effective, screwing effect and torque is desired (G5);
- to avoid an increase of CW rotation angle to prevent screwing effects and unload torque increases (G4);
- to reduce the range linked to a low CCW rotation angle when a low unload torque is desired (G1 and G2).

Therefore, our results allow to conclude on a direct influence of rotation angles on the mechanical behavior of endodontic instruments. The implementation of these data could improve the mechanical behavior of reciprocating instruments. Endodontic motors monitoring torque and pressure in real time could be a solution to adapt kinematic and mechanical behavior of instruments according to the different encountered clinical situations.

## Data Availability

The data that support the findings of this study are available from the corresponding author upon reasonable request.
